# A Novel Missense Mutation of the *DDHD1* Gene Associated with Juvenile Amyotrophic Lateral Sclerosis

**DOI:** 10.3389/fnagi.2016.00291

**Published:** 2016-12-06

**Authors:** Chujun Wu, Dongsheng Fan

**Affiliations:** Department of Neurology, Peking University Third HospitalBeijing, China

**Keywords:** amyotrophic lateral sclerosis, *DDHD1* gene, hereditary spastic paraplegia, juvenile, mutation

## Abstract

**Background:** Juvenile amyotrophic lateral sclerosis (jALS) is a rare form of ALS with an onset age of less than 25 years and is frequently thought to be genetic in origin. *DDHD1* gene mutations have been reported to be associated with the SPG28 subtype of autosomal recessive HSP but have never been reported in jALS patients.

**Methods:** Gene screens for the causative genes of ALS, HSP and CMT using next-generation sequencing (NGS) technologies were performed on a jALS patient. Sanger sequencing was used to validate identified variants and perform segregation analysis.

**Results:** We identified a novel c.1483A>G (p.Met495Val) homozygous missense mutation of the *DDHD1* gene in the jALS patient. All of his parents and young bother were heterozygous for this mutation. The mutation was not found in 800 Chinese control subjects or the database of dbSNP, ExAC and 1000G.

**Conclusion:** The novel c.1483A>G (p.Met495Val) missense mutation of the *DDHD1* gene could be a causative mutation of autosomal recessive jALS.

## Introduction

Juvenile amyotrophic lateral sclerosis (jALS) is a rare form of ALS with an onset age of less than 25 years and is thought to more frequently have a genetic origin than the adult-onset forms. Juvenile ALS is a clinically and genetically heterogeneous disease. Mutations in *ALS2, SETX* and *SPG11* are known to cause familial jALS with slow disease progression (Orban et al., 2007). In recent literatures, *SIGMAR1* have been reported to be a new causative gene of autosomal recessive jALS (Al-Saif et al., 2011; Ullah et al., 2015). However, in sporadic juvenile ALS patients, mutations in *Fus* are the most frequent genetic cause (Hübers et al., 2015; Zou et al., 2016). On the contrary to slow progression in familial jALS, sporadic jALS patients with *FUS* or *SOD1* mutations experienced aggressive progression and short survival times (Zou et al., 2016). Some of jALS causative genes have also been reported in other diseases, such as *ALS2* and *SPG11* causing hereditary spastic paraplegia (HSP) as well (Eymard-Pierre et al., 2002; Klebe et al., 2015). In this report, we describe a jALS patient with a novel missense mutation in the *DDHD1* gene, which is a member of the intracellular phospholipase A1 gene family and involved in the regulation of mitochondrial function. Associations have been reported between mutations in the *DDHD1* gene and the SPG28 subtype of autosomal recessive HSP but have never been reported in jALS patients.

## Materials and methods

### Subjects

**Figure 1:**
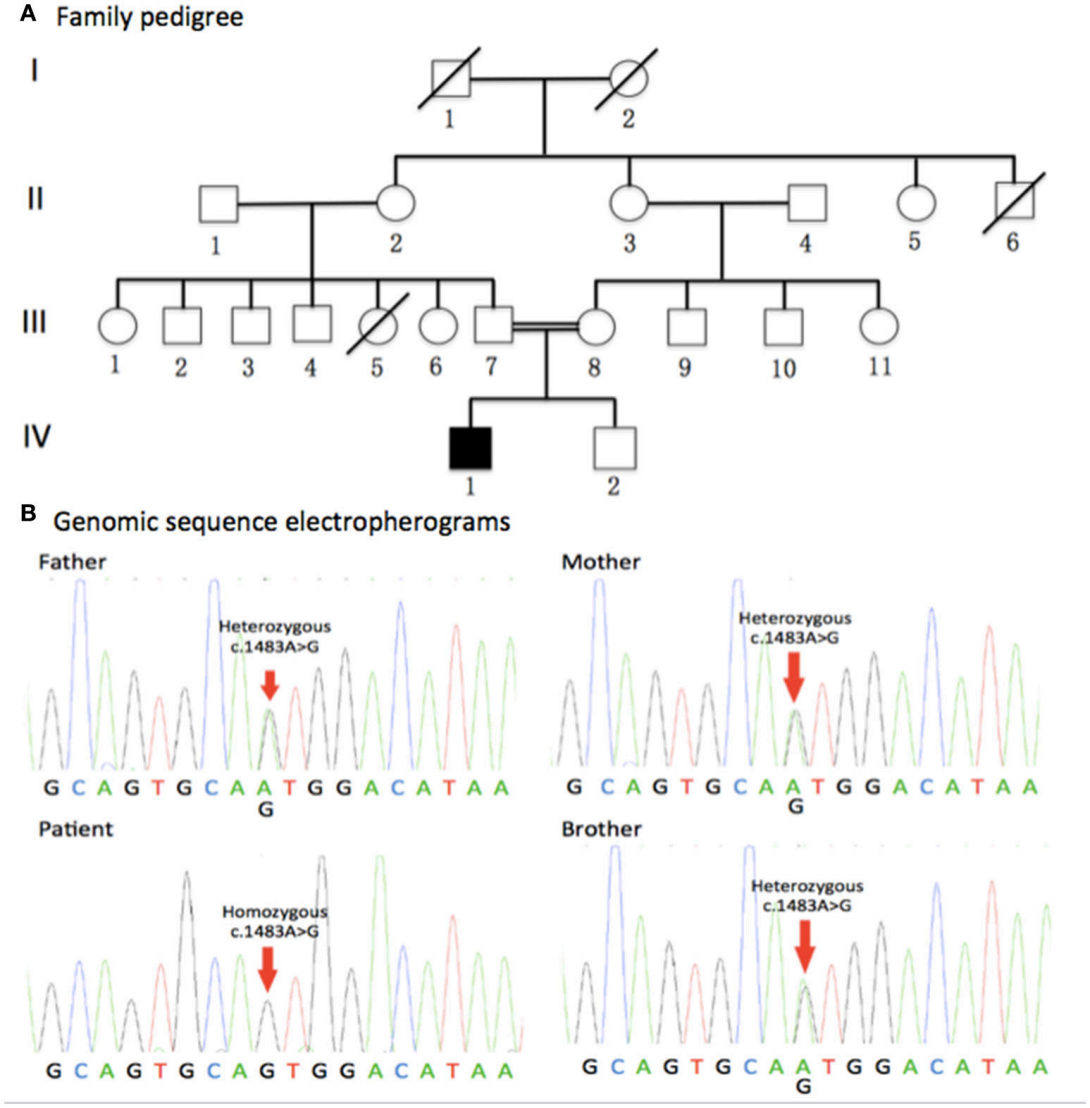
**Family pedigree and genomic sequence electropherograms. (A)** Family pedigree. The jALS patient was born to consanguineous Chinese parents. All of his parents and young brother were healthy. Males and Females are represented as squares and circles, respectively. The filled symbol represents the affected patient while unaffected individuals are represented by clear symbols. Crossed circles or squares represent deceased individuals. A double line indicates consanguineous mating. **(B)** Genomic sequence electropherograms. The jALS patient carried a novel homozygous c.1483A>G (p.Met495Val) missense mutation of the *DDHD1* gene, which was not detected in 800 healthy unrelated Chinese individuals. All of his parents and young brother were heterozygous for this mutation. Pedigree analysis suggested that the disease is consistent with autosomal recessive inheritance.

The pedigree for the family is presented in the Figure 1A. The clinical characteristics of the patient will be discussed in results section.

### Ethics statement

The institutional ethics committee of Peking University Third Hospital approved this study (IRB00006761). Written, informed consent was obtained from each participant.

### Genetic analysis

We obtained blood sample from affected and unaffected subjects in the pedigree (III-7, III-8, IV-1, IV-2). Next generation sequencing (NGS) was performed on an Illumina GAIIx platform to screen for variations in the patient, which covers the coding exons and flanking intronic sequences of 190 causative genes of ALS, HSP, and Charcot-Marie-Tooth disease (CMT) (Supplementary file). Identified variants in NGS were validated by Sanger sequencing. In order to perform segregation analysis, all of the patients' parents and young brother were screened for the identified variants using Sanger sequencing.

## Results

### Clinical features

The patient (IV-1) was a 24-year-old male who developed walking difficulties due to leg weakness beginning at 16 years of age and exhibited atrophy of the bilateral first interosseous muscles accompanied by a mild weakness in the hands 1 year later. These symptoms have progressed slowly. Besides, he didn't complain of any sensory abnormality. He was born to consanguineous Chinese parents. His parents and younger brother were clinically healthy. All of them didn't present any weakness or atrophy of muscle.

Neurologic examinations were performed for the patient (IV-1), his parents (III-7, III-8) and young brother (IV-2). In the patient, examinations revealed a steppage gait, atrophy of the bilateral interosseous and thenar muscles with a split-hand sign, mild weakness in the hands and lower limbs, hyperreflexia in all limbs with positive bilateral Babinski signs and Hoffmann signs, and disappearance of abdominal reflexes. Sensation and coordination were normal. As for the patients' parents and young brother, neurological examinations showed no muscle atrophy, normal tendon reflex and negative pathological reflex.

Needle EMG was performed on the patient. The results showed neurogenic changes, including fibrillation potentials and positive sharp waves, in four regions (brainstem, cervical, thoracic, and lumbosacral spinal cord). Nerve conduction studies revealed decreases in the amplitude of compound motor and sensory action potentials with an almost normal nerve conduction velocity. The structure brain and cervical MRIs were normal. Upon ^1^H-MRS examination, no pathological lactate accumulation was found in the cerebrospinal fluid of lateral ventricles.

### Genetic results

Gene screens for the causative genes of ALS, HSP, and CMT using NGS technologies identified a novel homozygous c.1483A>G (p.Met495Val)(RefSeq NM_001160147.1) missense mutation of the *DDHD1* gene in the patient (Figure 1B), which was verified by Sanger sequencing. Meanwhile, Sanger sequencing revealed all of his parents and young brother were heterozygous for this mutation (Figure 1B). The mutation was not detected in 800 healthy unrelated Chinese individuals by whole exome sequencing or NGS.

## Discussion

*DDHD1*, also known as *SPG28* or *PA-PLA1*, is a member of the intracellular phospholipase A1 gene family. The protein encoded by the *DDHD1* gene is a cytosolic protein with some mitochondrial localization and is involved in the regulation of mitochondrial dynamics via phosphatidic acid (PA) (Baba et al., 2014). The PA on the surface of mitochondria is known to regulate mitochondrial fusion. *DDHD1* is the first identified intracellular phospholipase A1 and preferentially hydrolyzes PA *in vitro*. The *DDHD1* pathogenic mutations cause reduced PA-PLA1 activity, and the resultant increased PA content on the surface of mitochondria might cause the impairment of mitochondrial fusion and lead to the dysfunction of mitochondria (Tesson et al., 2012). The previous study has showed the ectopic expression of *DDHD1* in HeLa cells induced mitochondrial fragmentation, whereas its depletion caused mitochondrial elongation. Gene disruption of *DDHD1* in mice caused sperm malformation due to mitochondrial organization defects (Baba et al., 2014). In patients harboring pathogenic *DDHD1* gene mutations, histochemical analyses in muscle showed mitochondrial alterations, and multiple mitochondrial DNA (mtDNA) deletions were evident (Mignarri et al., 2016). Besides, mitochondrial respiration rate, total cellular and mitochondrial ATP content were found to be significantly lower in lymphoblast from SPG28 patients (Tesson et al., 2012). In brain ^1^H-MRS analysis, a mild pathological accumulation of lactate in the cerebrospinal fluid was detected in a SPG28 patient with 20 years of disease duration, (Liguori et al., 2014) but not other three patients with shorter disease duration. (Liguori et al., 2014; Mignarri et al., 2016)

In previous studies, 8 mutations of the *DDHD1* gene have been reported in autosomal recessive HSP patients, including three nonsense mutations, three frame-shift mutations and two mutations affecting the mRNA splicing site (Tesson et al., 2012; Liguori et al., 2014; Mignarri et al., 2016; Miura et al., 2016). All of these mutations predict changes in the protein translation of the DDHD domain, which is responsible for the phospholipase activity of the *DDHD1* protein, thereby leading to a loss of function in the protein and finally mitochondrial dysfunction.

**Figure 2:**
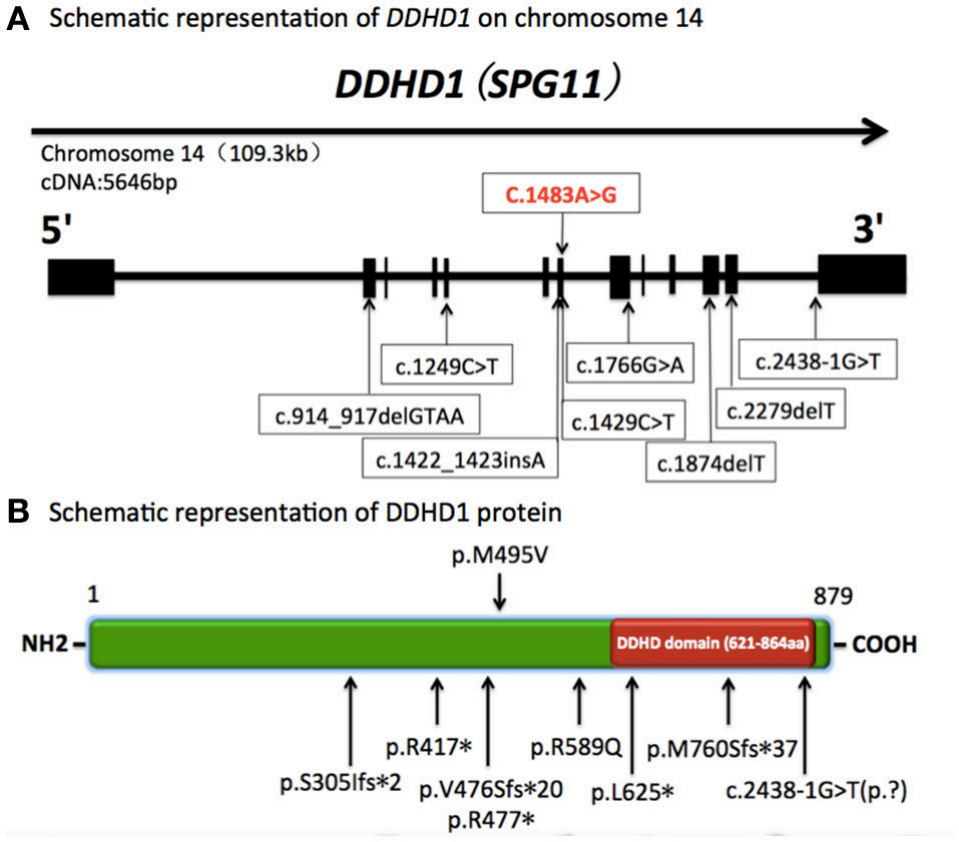
**Schematic representation of *DDHD1* on chromosome 14 and DDHD1 protein. (A)** Schematic representation of *DDHD1* on chromosome 14 (RefSeq NM_001160147.1). The black boxes represent coding exons. The upper row shows the novel missense mutation c.1483A>G (p.Met495Val) in our report, and the lower row shows the mutations in previous reports.c.1249C>T, c.1766G>A, c.1874delT, c.2438-1G>T (RefSeq NM_030637.2) (Tesson et al., 2012). c.1422_1423insA, c.2279delT (RefSeq NM_030637.2) (Liguori et al., 2014). c.1429C>T (RefSeq NM_001160148) (Mignarri et al., 2016). c.914_917delGTAA (RefSeq NM_030637.2) (Miura et al., 2016). **(B)** Schematic representation of DDHD1 protein (RefSeq NP_001153619.1). The red box represents DDHD domain in the C-terminus. The upper row shows the novel p.M495V mutation identified in this study. The known mutations are indicated below. p.R417^*^, p.R589Q, p.L625^*^, c.2438-1G>T(p.?)(RefSeq NP_085140.2) (Tesson et al., 2012). p.V476Sfs^*^20, p.M760Sfs^*^37 (RefSeq NP_085140.2) (Liguori et al., 2014). p.R477^*^ (RefSeq NP_001153620.1) (Mignarri et al., 2016). p.S305Ifs^*^2 (RefSeq NP_085140.2) (Miura et al., 2016).

In our report, the patient carried a novel homozygous c.1483A>G (p.Met495Val) missense mutation located in exon 7 of the *DDHD1* gene (Figure 2). His parents and younger brother were heterozygous for this mutation. Pedigree analysis suggested that the disease is consistent with autosomal recessive inheritance. The mutation was not detected in 800 healthy unrelated Chinese individuals, suggesting that the mutation is not present in the normal Chinese population.

**Figure 3:**
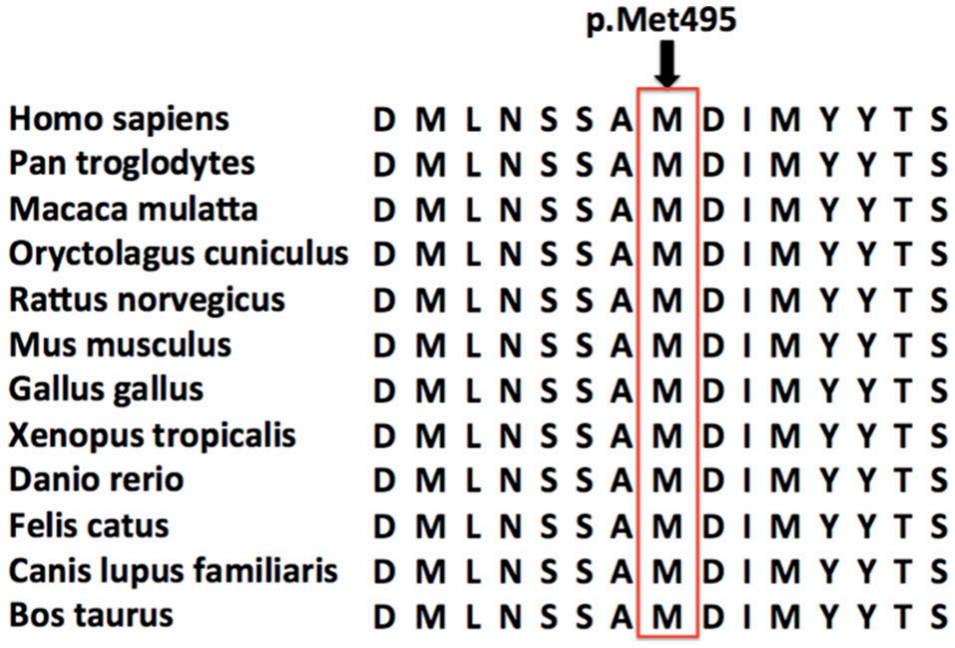
**Conservation of the amino acid affected by the missense mutation**. Amino acid sequence alignments around the single amino acid affected by the novel *DDHD1* gene mutation are shown for selected species. The amino acid affected by the novel p.Met495Val missense mutation is highlighted by a red rectangle. This novel mutation was located in a very conserved domain, and was predicted to be disease causing by MutationTaster, possibly damaging by Polyphen-2 and affecting protein function by SIFT.

Furthermore, this new mutation, located in a conserved domain (Figure 3), was not found in dbSNP, ExAC or 1000G database and was predicted to be disease causing by MutationTaster, possibly damaging by Polyphen-2 and affecting protein function by SIFT. So we concluded this variant was a pathogenic mutation rather than a polymorphism.

Mitochondrial dysfunction is one of the important pathophysiological mechanisms in ALS (Mancuso and Navarro, 2015). In our report, the novel homozygous missense *DDHD1* mutation might cause jALS via mitochondrial dysfunction. Unlike the previously reported mutations, the new mutation may not change the protein primary structure but rather the three-dimensional structure of the DDHD domain, ultimately causing a loss of function in the protein. However, further functional studies are needed to confirm mitochondrial dysfunction in ALS patients with *DDHD1* gene mutations.

Patients with *DDHD1* gene mutations have presented pure or mildly complicated HSP in previous studies (Bouslam et al., 2005; Tesson et al., 2012; Liguori et al., 2014; Mignarri et al., 2016; Miura et al., 2016). Tongue fasciculation with wasting have been reported previously, (Liguori et al., 2014) indicating that *DDHD1* gene mutations may influence lower motor neuron (LMN) function. In this patient, muscle atrophy of hands with a split-hand sign was apparent, and needle EMG confirmed neurogenic damage in four regions. We believe that the decreased amplitude of compound motor action potentials was the result of severe LMN damage. Apart from motor system involvement, subclinical sensory axonal neuropathy was detected in this patient. In previous reports, clinical and subclinical sensory defects had been reported in HSP patients with *DDHD1* gene mutations, which reflected *DDHD1* gene mutations might cause sensory defects (Bouslam et al., 2005; Liguori et al., 2014). Moreover, subclinical sensory abnormalities, peripheral as well as central levels, could be found in ALS patients who didn't have other known potential causes of polyneuropathy, such as diabetes mellitus (Pugdahl et al., 2007; Iglesias et al., 2015; Isak et al., 2016). A multicenter study reported 22.7% patients with ALS had sensory nerve action potentials (SNAPs) abnormalities in at least one nerve (Pugdahl et al., 2007). Although pathophysiological mechanisms underlying sensory abnormalities remains to be further clarified, these findings reflected that ALS might be a multi-systemic disorder involving other systems than motor. In the revision of the EI Escorial criteria 2015, sensory impairment does not exclude ALS diagnosis. Besides, UMN and LMN impairment, rather than subclinical sensory impairment, was the dominant presentation in this patient. Finally, the patient was diagnosed with lab-supported probable jALS. Considering the clinical history of this patient and previously reported cases, we believe that jALS patients with *DDHD1* mutations could have slow disease progression, similar to that of other familial jALS patients.

In summary, this is the first case report about the relationship between *DDHD1* gene mutations and jALS. Besides of dominant motor system involvement, subclinical sensory impairment was detected in this jALS patient. All of these findings reflected the clinically and genetically heterogeneity of jALS and further proved the genetic overlap between HSP and jALS. Meanwhile, further functional studies are needed to confirm the effects of *DDHD1* gene mutations in ALS pathogenesis.

## Conclusion

Juvenile ALS is a clinically and genetically heterogeneous disease. As described in our report, we discovered a novel c.1483A>G (p.Met495Val) missense mutation of the *DDHD1* gene that could be a causative mutation of autosomal recessive jALS.

## Author contributions

DF conceived this study and provided financial support; CW and DF performed the experiments, analyzed the data, and wrote the manuscript.

## Funding

This study was supported by the National Natural Science Foundation of China (81030019).

### Conflict of interest statement

The authors declare that the research was conducted in the absence of any commercial or financial relationships that could be construed as a potential conflict of interest.
