# Supplementary material for: A Novel Missense Mutation of the DDHD1 Gene Associated with Juvenile Amyotrophic Lateral Sclerosis
Source: Front Aging Neurosci. 2016 Dec 6;8:291. doi: 10.3389/fnagi.2016.00291 (PMC5138217; doi:10.3389/fnagi.2016.00291)
Supplement: Supplementary file 1 [file DataSheet1.DOC]

**Appendix**

Next generation sequencing (NGS) technologies were used to screen for causative genes of amyotrophic lateral sclerosis (ALS), hereditary spastic paraplegia (HSP) and Charcot-Marie-Tooth disease (CMT).

Candidate genes for ALS (78):

ALAD, ALS2, ANG, APEX1, APOE, ARHGEF28, BCL11B, BCL6, CDH13, CDH22, CHGB, CHMP2B, CHRNA4, CNTN6, CRIM1, CRYM, DAO, DCTN1, DIAPH3, DOC2B, DPP6, DPYSL3, ELP3, EPHA4, ERBB4, EWSR1, FEZF2, FGGY, FIG4, FUS, GRB14, GRN, HFE, HNRNPA1, HNRNPA2B1, ITPR2, KIFAP3, LUM, MAPT, NEFH, NETO1, NIPA1, OGG1, OMA1, OPTN, PARK7, PCP4, PFN1, PON1, PON2, PON3, PRPH, RAMP3, SETX, SIGMAR1, SLC52A2, SLC52A3, SMN1, SOD1, SOX5, SPAST, SPG11, SQSTM1, SRCAP, SS18L1, SUSD1, SYT9, TAF15, TARDBP, TRPM2, UBQLN1, UBQLN2, UNC13A, VAPB, VCP, VEGFA, ZFP64, ZNF512B

Candidate genes for HSP and CMT (112):

AARS, AIFM1, AMPD2, AP4B1, AP4E1, AP4M1, AP4S1, ARL6IP1, ARSI, ATL1, ATP7A, ATRX, B4GALNT1, BICD2, BSCL2, C12orf65, C19orf12, CCT5, CTDP1, CYP2U1, CYP7B1, DDHD1, DDHD2, DHTKD1, DNAJB2, DNM2, DNMT1, DYNC1H1, EGR2, ENTPD1, ERLIN1, ERLIN2, FA2H, FAM134B, FBLN5, FGD4, FLRT1, GARS, GBA2, GDAP1, GJB1, GJC2, GNB4, HARS, HINT1, HK1, HOXD10, HSPB1, HSPB3, HSPB8, HSPD1, IGHMBP2, IKBKAP, INF2, KARS, KIAA0196, KIAA0415, KIF1A, KIF1B , KIF1C, KIF5A, L1CAM, LITAF, LMNA, LRSAM1, MARS, MED25, MFN2, MPZ, MTMR2, MYH14, NDRG1, NEFL, NT5C2, NTRK1, OPA1, PDK3, PGAP1, PLEKHG5, PLP1, PMP22, PNPLA6, PRNP, PRPS1, PRX, RAB3GAP2, RAB7A, REEP1, RTN2, SACS, SBF1, SBF2, SCN9A, SH3TC2, SLC16A2, SLC33A1, SLC5A7, SPG20, SPG21, SPG7, SPTLC1, SPTLC2, TFG, TRIM2, TRPV4, USP8, WDR48, WNK1, YARS, ZFR, ZFYVE26, ZFYVE27
